# Comparison of laparoscopic selective colectomy based on barium-strip examination and subtotal colectomy for adult slow-transit constipation

**DOI:** 10.1093/gastro/goz020

**Published:** 2019-07-16

**Authors:** Zhao Hui Zhong, Shen Yang, Yong Zhao, Yuan Wang, Wei Dong Yong, Ling Ling Zhang, Qiu Sheng Wang, Xun Huang

**Affiliations:** 1 General Surgery Department, Peking University People’s Hospital, Beijing, P.R. China; 2 Insitute of Laboratory Animal Science, Chinese Academy of Medical Sciences & Peking Union Medical College, Beijing, P.R. China

**Keywords:** slow-transit constipation, laparoscopic selective colectomy, laparoscopic subtotal colectomy, barium strip, quality of life

## Abstract

**Background:**

Surgical management of adult slow-transit constipation (ASTC) can be effective for patients with intractable symptoms. This study aimed to evaluate whether barium-strip examination and selective colectomy improved post-operative outcomes in ASTC patients in comparison with subtotal colectomy.

**Methods:**

A retrospective cohort study of 53 cases with refractory ASTC was conducted between June 2008 and June 2014. Patients were evaluated by the barium-strip technique, colonoscopy, defecography and anorectal manometry. Patients in the standard group underwent laparoscopic subtotal colectomy and patients in the laparoscopic selective colectomy (LSC) group underwent LSC at the precise location identified by barium strip. Spontaneous bowel movements, the Wexner Constipation Scale and the Gastrointestinal Quality of Life Index (GIQLI) were assessed post-operatively at 3, 6, 12 and 24 months.

**Results:**

A total of 49 patients were included in the analysis. The median follow-up was 37 months (range, 26–60 months). The mean post-operative hospital stay was 12 days and similar between groups (*P *=* *0.071). The length of colon resection, operative time and intra-operative blood loss were reduced in the LSC group (all *P *<* *0.05). No major complications occurred. A similar number of patients (24 in the standard group and 25 in the LSC group) exhibited hypoganglionosis or aganglionosis in the colon-wall muscle layer (*P *=* *0.986). Although there were no significant differences in post-operative spontaneous bowel movements and the Wexner Constipation Scale between the two groups, the mean GIQLI of the LSC group was significantly higher at 3, 6 and 24 months post-operatively (all *P *<* *0.05).

**Conclusions:**

LSC based on barium-strip examination is an appropriate modality for treating ASTC.

## Introduction

Adult slow-transit constipation (ASTC) is defined as prolonged transit time through the colon. Patients with ASTC have normal resting colonic motility, but do not have the increase in peristaltic activity that should occur after meals [1]. ASTC is a benign gastrointestinal disease, though it is commonly associated with symptoms that include an infrequent ‘call to stool’, bloating and abdominal discomfort. These symptoms interfere with the patient’s quality of life. ASTC can result from several factors, including the person’s lifestyle (e.g. low-fiber diet, inadequate fluid intake), ingestion of certain drugs or lesions causing slow colon motility. Therefore, the first approach for treating ASTC is lifestyle changes, including increased fiber and fluid in the diet and increased exercise. If this fails to ameliorate the symptoms, ASTC is commonly managed medically, initially through administration of laxatives [2]. Some success in treating symptoms has also been found with the addition of probiotic intake in the diet. However, some patients are administered other treatments to relieve their symptoms, including prucalopride, linaclotide, biofeedback or sacral-nerve stimulation [3, 4].

Surgical options are available for a small proportion of patients with intractable symptoms and poor quality of life. One such surgery is total abdominal colectomy with ileorectal anastomosis [5]. Surgery is also required in some patients to correct anatomical problems, such as stenotic diverticulitis or an outlet problem. A century ago, Sir Arbuthnot Lane was the first to describe the use of colectomy to treat chronic constipation. Since then, various combinations of surgical procedures have been used [6] and procedures involving subtotal colectomy or segmental colectomy have been shown to be effective alternatives to total colectomy [7–9]. Outcomes from surgical treatment are generally positive, but some patients experience no improvement in symptoms or adverse events such as diarrhea [10]. With precise diagnostic efforts, the extent of ASTC surgical procedures may be kept to a minimum. In recent years, several colorectal procedures have been increasingly performed laparoscopically, with the inherent advantages of reduced post-operative pain and ileus, superior cosmetic results, fewer post-operative adhesions, reduced hospital stay and earlier return to work [11, 12]. The use of laparoscopy to perform subtotal colectomy has been reported to be feasible and safe [13–15].

Most studies regarding the surgical management of refractory chronic constipation have been conducted in Western countries [16, 17]. Few studies have addressed the need for surgery and/or therapeutic outcomes in East-Asian populations, where there is enormous diversity in the ethnic population in terms of dietary and bowel habits. Thus, the indications for surgery may also be different [18, 19].

We hypothesized that finding and resecting a precisely located segment of the colon is likely to be a crucial measure for improving surgical outcomes for ASTC. Therefore, we recently began localizing lesions in the colon using a barium-strip examination prior to laparoscopic subtotal colectomy and laparoscopic selective colectomy (LSC). The aim of this study was to evaluate the post-operative outcomes of LSC based on barium-strip examination in ASTC patients. Furthermore, we compared the surgical outcomes between the selective and subtotal procedures.

## Materials and methods

### Study design

This was a retrospective cohort study of 53 cases with refractory ASTC conducted between June 2008 and June 2014 at the Department of General Surgery in the Peking University People’s Hospital (PKUPH). The PKUPH Ethic and Medical Research Committee approved the study. Consent for each surgery was obtained from all patients after informing them of the nature of the disease, treatment options and possible outcomes.

### Patients

Patients with ASTC were pre-operatively considered for surgery and inclusion in the study using the following criteria: (i) chronic (over 60 months), severe (Wexner Constipation Scale [WCS] >15 [20]) and incapacitating symptoms where quality of life is severely jeopardized; (ii) aged from 18 to 80 years, without gastric function or small-bowel motility disorders; (iii) meeting the Rome III criteria for chronic constipation; (iv) conservative treatment with diet, laxatives, enemas and biofeedback had been tried over the past 2 years without success; (v) completing at least 2 years of post-operative follow-up. Exclusion criteria included patients with (i) contraindication to general anesthesia; (ii) immunocompromised status; (iii) physical or psychological problems precluding data collection; (iv) inflammatory bowel disease or septic conditions of the anorectum.

All patients initially presented to our department, where each was evaluated clinically with a detailed history and physical examination. The presence of ASTC was defined as irritable bowel syndrome with constipation when it met the following criteria: fewer than three spontaneous bowel movements (SBMs) per week and one or more of the following symptoms for at least 12 weeks during the preceding 12 months: (i) straining during ≥25% of defecations; (ii) lumpy or hard stools in ≥25% of defections; (iii) sensation of incomplete evacuation in ≥25% of defecations; (iv) mean score of ≥2.0 for daily non-menstrual abdominal pain or discomfort on a five-point scale ranging from 1 (indicating no pain) to 5 (indicating severe pain). Surgery was considered when at least one of the following criteria was present [21]: (i) patient required excessive cathartics because of a failure of osmotic laxatives to work, thus requiring stimulant laxatives or enemas on a regular basis; (ii) patient was not responding to cathartics (fewer than three SBMs per week and with unusually prolonged straining despite taking a high-dose laxative); (iii) fecal impaction and rectal prolapse was detected on rectal examination; (iv) gastrointestinal transit test showed slow colonic transit; (v) gastrointestinal organic disease was excluded by colonoscopy or barium enema.

### Indicators and evaluations

**Figure 1.**
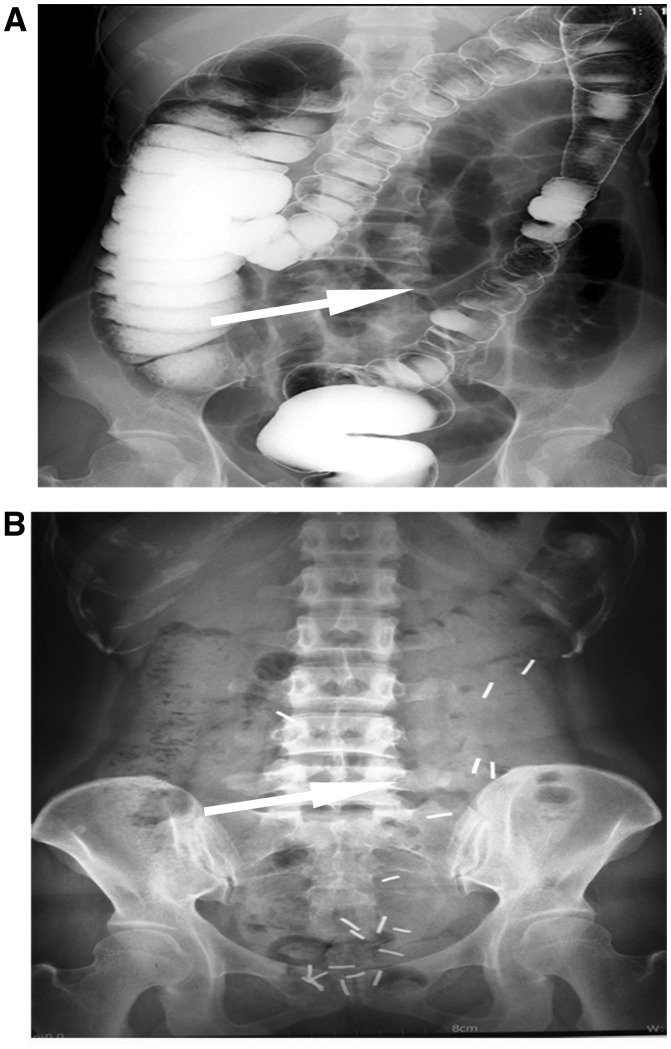
Pre-operative barium-enema examination (**A**) and barium-strip examination (**B**) of a patient with slow-transit constipation who underwent laparoscopic selective colectomy

All patients were evaluated based on a standard protocol using the barium-strip technique (Figure 1), colonoscopy, and defecography [21]. Systemic causes were excluded by determining the serum thyroxine, thyroid-stimulating hormone and postprandial blood glucose levels in all cases.

Colonic transit time and colon-lesion localization were determined using locally manufactured radiopaque barium-strip markers in gelatin capsules. The subjects were asked to ingest 20 barium-strip markers at a time (10 markers in each capsule). Subsequently, abdominal radiographs were obtained at 36, 48 and 72 hours after ingestion. We defined a positive colonic transit test as having greater than 20% of the barium strip still present in the colorectum after 96 hours. Colonic distribution of markers indicated the colonic-inertia segment. Anal manometry was conducted to assure no outlet obstruction. A barium enema was used to make certain that no mechanical obstruction accounted for the constipation.

Outpatient interview 1 week before operation assessed SBMs, WCS and Gastrointestinal Quality of Life Index (GIQLI) [22]. SBMs were defined as passage of stool without the use of laxatives, enemas or digital evacuation.

### Surgical interventions and follow-up

All eligible patients were divided into a laparoscopic subtotal colectomy group (standard group) and an LSC group. All resection procedures were performed according to the indications for surgery. Patients in the standard group underwent laparoscopic subtotal colectomy, as previously described. Patients in the LSC group underwent LSC, since the lesion in the colon had been precisely localized by pre-operative barium-strip examination. The resected region included the prolonged transit segment on the barium-strip test and the excision border lacked barium strips.

The patients were closely monitored during the perioperative period for complications and were then followed up in an outpatient clinic, where details regarding SBMs were documented weekly. Outpatient and telephone interviews were used to assess SBMs, WCS and GIQLI post-operatively at 3, 6, 12 and 24 months, according to the criteria given above.

### Statistical analysis

Analyses were performed using the SPSS statistical software package (version 18.0; SPSS Inc., Chicago, IL, USA). Quantitative variables were summarized as mean with standard deviation or median with interquartile range (IQR) and comparisons between groups were made by using independent samples *t*-test or paired *t*-test. Categorical variables were summarized as frequency with percentage and comparisons between groups were performed by using Chi-square test or Fisher’s exact test. A *P*-value of less than 0.05 was considered statistically significant.

## Results

### Baseline information

A total of 53 patients were treated surgically for ASTC between June 2008 and June 2014, among whom 2 patients were excluded for having orthopedic surgery and 2 failed to follow up. The characteristics of the final cohort of 49 patients are shown in Table 1. There were 7 males and 17 females in the standard group, with a mean age of 40.6 years (range, 21–80 years). In the LSC group, there were 10 males and 15 females, with a mean age of 42.0 years (range, 20–79 years). Pre-operatively, conservative treatment with diet, laxatives, enemas and biofeedback had been attempted unsuccessfully over the past 2 years in all patients. All of the patients complained of constipation, the sensation of incomplete bowel evacuation and intermittent mild abdominal pain for a long period of time. The median duration of these symptoms was 60 months (range, 36–480 months). The median duration of medical management was 48 months (range, 36–480 months). The durations of both symptoms and medical treatment were similar between the two groups.

**Table 1.** Baseline characteristics of 49 patients with slow-transit constipation

Item	Standard group (*n *=* *24)	LSC group (*n *=* *25)	*P*-value
Female, *n* (%)	17 (70.8)	15 (68.0)	0.830
Mean age, years	40.6 ± 19.0	42.0 ± 18.6	0.220
Pre-operative evaluation			
Mean WCS	18.08 ± 2.62	18.16 ± 2.15	0.911
Mean SBMs	1.07 ± 0.40	0.98 ± 0.27	0.348
Mean GIQLI	84.42 ± 7.86	80.00 ± 7.82	0.089

LSC, laparoscopic selective colectomy; WCS, Wexner Constipation Scale; SBMs, spontaneous bowel movements; GIQLI, Gastrointestinal Quality of Life Index.

### Surgical results

The mean length of resected colon (65 ± 29 vs 122 ± 23 cm, *P *<* *0.05), operative time (195 ± 54 vs 249 ± 53 minutes, *P *<* *0.05) and intra-operative blood loss (58 ± 20 vs 100 ± 61 mL, *P *<* *0.05) were greatly reduced in the LSC group compared to the standard group (Table 2). The mean post-operative hospital stay in the LSC group seemed to be shorter than that in the standard group (10.5 ± 4.0 vs 12.6 ± 3.9 days), but the difference did not reach statistical significance (*P *=* *0.071). Twenty-two patients in the standard group and 24 in the LSC group showed a definite improvement in symptoms, with daily evacuations.

**Table 2.** Perioperative characteristics of 49 patients with slow-transit constipation

Item	Standard group (*n *=* *24)	LSC group (*n *=* *25)	*P*-value
Mean length of resection colon, cm	122 ± 23	65 ± 29	<0.001
Mean operative time, minutes	249 ± 53	195 ± 54	<0.001
Mean intra-operative blood loss, mL	100 ± 61	58 ± 20	<0.001
ASA score, *n* (%)			0.914
1	14 (58.3)	16 (64.0)	
2	8 (33.3)	7 (28.0)	
3	2 (8.3)	2 (8.0)	
Pathologic finding, *n* (%)			0.986
Normal	6 (25.0)	6 (24.0)	
Hypoganglionosis	10 (41.7)	11 (44.0)	
Aganglionosis	8 (33.3)	8 (32.0)	
Mean length of hospital stay, days	12.6 ± 3.9	10.5 ± 4.0	0.071
Anastomotic leakage, *n* (%)	3 (12.5)	3 (12.0)	0.881
Post-operative ileus, *n* (%)	3 (12.5)	3 (12.0)	0.881

LSC, laparoscopic selective colectomy; ASA, American Society of Anesthesiologists.

**Figure 2.**
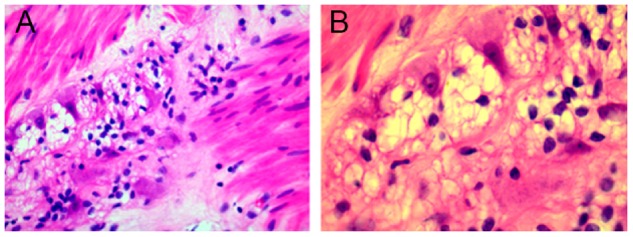
Pathological findings in resected colon show hypoganglionosis in the colon-wall muscle layer. (**A**) Hematoxylin-eosin staining,×200; (**B**) hematoxylin-eosin staining,×400.

No major intra-operative complications occurred and no patients died of perioperative complications. Post-operative pathological examination revealed that 18 patients in the standard group and 19 patients in the LSC group exhibited hypoganglionosis or aganglionosis in the colon-wall muscle layer (*P *=* *0.986; Table 2). Additionally, the region of the colon that had been resected showed a lack of normally distributed neurons (Figure 2).

### Long-term outcomes

The median follow-up was 37 months (range, 26–60 months) and no major post-operative complications occurred during follow-up. Four patients in the standard group and three patients in the LSC group presented with slightly watery diarrhea 1 month post-operatively, though the symptoms improved after 6 months. One patient in the standard group presented with abdominal pain 6 months after surgery, which resolved following dilation of a stenotic anastomosis.

**Figure 3.**
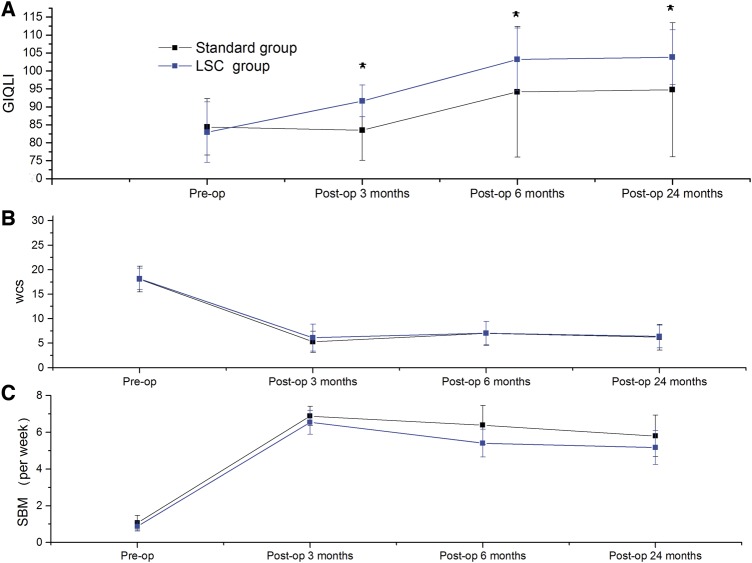
Comparisons of the Gastrointestinal Quality of Life Index (GIQLI) (**A**), Wexner Constipation Scale (WCS) (**B**) and spontaneous bowel movement (SBM) (**C**) between the standard group and LSC group (*P <* *0.05). Pre-op, pre-operative; Post-op, post-operative.

Surgical treatment improved patients’ outcomes in both groups, including increased SBMs and GIQLI and decreased WCS (Figure 3). Although there were no significant differences in post-operative SBMs and WCS between the two groups, the mean GIQLI of the LSC group was significantly higher than that of the standard group at 3 months (90.08 ± 5.15 vs 83.5 ± 8.37, *P *=* *0.002), 6 months (103.44 ± 10.13 vs 94.17 ± 18.18, *P *=* *0.032) and 24 months (104.16 ± 8.82 vs 94.75 ± 18.64, *P *=* *0.028) post-operatively.

## Discussion

The aim of this study was to evaluate whether barium-strip examination and selective colectomy improved post-operative outcomes in ASTC patients in comparison with subtotal colectomy. We selected subjective patient reports of bowel function (i.e. the WCS) to determine constipation and the GIQLI to assess bowel function. All of the patients in this study experienced difficulty in their daily lives because of symptoms associated with disorderly bowel function. Patients were also at risk of developing colorectal tumorigenesis. In some of the most severe cases in our study group, these symptoms led to insufficient nutritional intake. Most of these patients were satisfied with the outcomes of the surgery. A total of 49 patients were included in the final analysis over a median follow-up of 37 months. The post-operative hospital stays were similar between the two groups, but the length of colon resection, operative time and operative bleeding were reduced in the LSC group. No major complications occurred in either group. A large number of patients in the standard (*n *=* *18) and LSC (*n *=* *19) groups exhibited hypoganglionosis or aganglionosis in the colon-wall muscle layer. The GIQLI of the LSC group was significantly higher than that of the standard group at 3, 6 and 24 months post-operatively.

It is well known that functional surgery represents an interesting challenge for gastrointestinal surgeons because of the intricacy of the underlying pathophysiological mechanisms, often resulting in extremely irregular and subjective responses. Therefore, addressing the risk–benefit balance is often stressful, with a difficult decision-making process for both the patients and the surgeons. To some extent, efforts have been concentrated on reducing the surgical burden by devising minimally invasive approaches for this type of operation, more so than for oncologic operations. The surgical treatment of ASTC described herein undoubtedly constitutes a prototype of these concerns and developments [23].

Ripetti *et al.* [19] have suggested that a malformation such as a dolichocolon is not directly responsible for slow transit, ascribing ASTC to a modification in peristalsis rather than to the length of the colon. Various surgical procedures for ASTC have been described, with total colectomy and ileorectal anastomosis being the most common (72%), followed by sigmoid colectomy (10%), subtotal colectomy with cecoproctostomy (6%) and left hemicolectomy (6%) [24]. Each of these procedures lacks precision, but ASTC studies using markers with defecography have offered the possibility of reaching a more accurate diagnosis and reducing the risk of complications [25].

Wang *et al.* [26] performed 31 selective colectomies based on pressure measurements of intelligent capsules and radiopaque particles to treat intractable transmission of chronic constipation and suggested that colon pressure-wave measurements and colon transportation tests could be instructive for selecting a surgical procedure. They found that selective colectomy resulted in less trauma and fewer post-operative complications. Less severe side effects have also been noted with segmental colonic resection in a study of 28 patients [27]. In this study, we determined the extent of ASTC by identifying the locations of the disease using the barium-strip technique.

Although patients in both groups improved significantly after surgery, our study indicated that the patients in the LSC group achieved greater potential benefits than the standard group in terms of post-operative GIQLI. When post-operative diarrhea occurred, it was present for only a short time, predominantly during the first 6 months, and did not require long-term therapy. With regard to constipation, the WCS was high pre-operatively but significantly declined during follow-up.

In patients with slow-transit constipation or obstructed defecation syndrome, the number of interstitial cells of Cajal was significantly decreased in all layers and the myenteric plexus displayed reduced ganglionic density and size compared with the normal colon [28, 29]. We carried out pathology examinations to localize distinct transitional zones in the segmented colon that required resection and observed the same results in surgical specimens. The percentages of hypoganglionosis and aganglionosis in the two groups were not significantly different. As the pathological examination showed, our ASTC patients had zones within the pathologically involved segments that lacked the normal distribution of neurons.

Some studies have questioned the feasibility of segmental colon resection and regarded the outcomes of laparoscopic colectomy as limited, with the possibility of recurrence. In the present study, we did not find any differences between the two groups in terms of gender, age, pathological characteristics, American Society of Anesthesiologists score, pre-operative WCS, SBMs or GIQLI. However, the reductions in operative time, intra-operative bleeding, length of colon resected and post-operative GIQLI, along with the lack of increased intra-operative complications or post-operative morbidity, suggest more potential advantages of LSC over laparoscopic subtotal colectomy.

This study has some limitations. The study design meant that the patients were not randomized into the groups; however, there were no significant differences in baseline characteristics between them. Factors that might potentially have an impact on the post-operative outcome were included in order to have two comparable groups, apart from the surgical approach used.

In conclusion, we recommend LSC based on barium-strip examination as the appropriate modality for treating ASTC. This technique has been shown to be a reliable, safe and efficacious approach with suitable outcomes for patients with ASTC.

## Authors’ contributions

Z.H.Z. and S.Y.: collection and assembly of data, provision of study material or patients, data analysis and interpretation, financial support. Z.Y. and Y.W.: collection and assembly of data. W.D.Y. and L.L.Z.: data analysis and interpretation, pathological experiments and interpretation, financial support. Q.S.W. and H.X.: provision of study material, administrative support. All authors read and approved the final manuscript.

## Funding

This study was supported by the National Science Foundation of China [No. 81700751] and the Scientific Research Foundation for the Returned Overseas Chinese Scholars [No. 2110000021].
